# Correlation between structure, chromaticity, and dielectric properties of calcium copper pyrophosphates, Ca_2−*x*_Cu_*x*_P_2_O_7_

**DOI:** 10.1038/s41598-022-11056-4

**Published:** 2022-04-27

**Authors:** Rattanai Baitahe, Chuchai Sronsri, Somphob Thompho, Kittichai Chaiseeda, Nattaya Montri, Banjong Boonchom

**Affiliations:** 1 Material Science for Environmental Sustainability Research Unit, School of Science, King Mongkut’s Institute of Technology Ladkrabang, Bangkok, 10520 Thailand; 2 Municipal Waste and Wastewater Management Learning Center, School of Science, King Mongkut’s Institute of Technology Ladkrabang, Bangkok, 10520 Thailand; 3 Pharmaceutical Research Instrument Center, Faculty of Pharmaceutical Sciences, Chulalongkorn University, Pathumwan, Bangkok, 10330 Thailand; 4 Organic Synthesis, Electrochemistry and Natural Product Research Unit (OSEN), Department of Chemistry, Faculty of Science, King Mongkut’s University of Technology Thonburi, Bangkok, 10140 Thailand; 5 Department of Plant Production Technology, School of Agricultural Technology, King Mongkut’s Institute of Technology Ladkrabang, Bangkok, 10520 Thailand

**Keywords:** Materials for optics, Structural materials, Techniques and instrumentation, Chemistry, Materials science, Nanoscience and technology

## Abstract

The solid-state reaction was employed to synthesize Ca_2−*x*_Cu_*x*_P_2_O_7_ by varying the mole ratio between Ca and Cu. The structure and crystallography of the pyrophosphate compounds were identified and confirmed by using X-ray diffraction (XRD). The Rietveld refinement method and the extended X-ray absorption fine structure (EXAFS) least-squares fitting technique were also applied to refine the sample crystal structure. The single phases of the obtained Ca_2_P_2_O_7_, CaCuP_2_O_7_, and Cu_2_P_2_O_7_ samples and the mixing phases of the obtained Ca_1.5_Cu_0.5_P_2_O_7_ and Ca_0.5_Cu_1.5_P_2_O_7_ samples were identified, and then only a single phase of the samples was subjected to structural and dielectrical analyses. The structural results exhibit the tetragonal crystal system with the *P4*_*1*_ space group for *β*-Ca_2_P_2_O_7_, the monoclinic crystal system with the *P2*_*1*_*/c* space group for CaCuP_2_O_7_, and the *C*2/*c* space group for *α*-Cu_2_P_2_O_7_. The dielectric constant (*ε*_r_) of the single metal pyrophosphates (Ca_2_P_2_O_7_ and Cu_2_P_2_O_7_) was higher than that of binary metal pyrophosphates (CaCuP_2_O_7_). The image sensor result of the Cu_2_P_2_O_7_ sample (*x* = 2.00) illustrated a yellowish-green color, while other compounds (*x* = 0.50−1.50) presented color tones that changed from blue-green to bluish-green. Raman and Fourier transform infrared (FTIR) spectrophotometers were employed to characterize and confirm the vibrational characteristics of the P_2_O_7_^4−^ group, which contains the O–P–O radical ([PO_2_]^-^) and the P–O–P bride ([OPO]^-^) and approximate M–O stretching modes. Furthermore, this work reports for the first time that the change in the crystal structure of Ca_2−*x*_Cu_*x*_P_2_O_7_ (i.e., bond angle of P−O−P in P_2_O_7_^4−^ and distortion phenomena in the *M*−O_6_ octahedral site) are cause the correlation between the structure, chromaticity, and dielectric properties of calcium copper pyrophosphates, Ca_2−*x*_Cu_*x*_P_2_O_7_.

## Introduction

Currently, metal phosphate materials show interesting properties because they are used in many applications. For example, they have been applied as microwave dielectric materials, corrosion-resistant coatings, biomedical cements, chelating agents, glass ceramics, and high-quality fertilizers^1–3^. Bian et al.^2^ reported that metal pyrophosphates (*M*_2_P_2_O_7_, *M* = divalent cations) show low-loss dielectric properties as well as a relatively low sintering temperature. When the ionic radius of *M* in the *M*_2_P_2_O_7_ structure is higher than 0.97 Å (*M* = Ca^2+^, Sr^2+^, Ba^2+^, Pb^2+^, Cd^2+^)^4^, *M*_2_P_2_O_7_ compounds crystallize in the dichromate (Cr_2_O_7_^2−^) form, in which a pair of P_2_O_7_^4−^ groups in eclipsed are the center of symmetry and bridging oxygen (O) atoms spread to each other. However, when the ionic radius of *M* is lower than 0.97 Å (*M* = Ni^2+^, Mg^2+^, Zn^2+^, Co^2+^, Cu^2+^, Mn^2+^), the *M*_2_P_2_O_7_ structure is a thortveitite type^5^ (scandium yttrium silicate (Sc,Y)_2_Si_2_O_7_ with the monoclinic crystal system, prismatic crystal class (2/m), and C2/m space group^6^). Based on this thortveitite structure, P_2_O_7_^4−^ groups occur in a staggered conformation. Moreover, compared to metal oxides (i.e., *M*O, *M* = divalent metals), thortveitite-type pyrophosphates, such as *α*-Cu_2_P_2_O_7_ and *α*-Mg_2_P_2_O_7_, exhibit a rather low sintering temperature. However, the single metal pyrophosphate groups, such as Cu_2_P_2_O_7_, Mg_2_P_2_O_7_, Zn_2_P_2_O_7_, and Co_2_P_2_O_7_, still show a phase transition with changing sintering temperature. Therefore, the first aim of this research is to modify the crystal structures of some metal pyrophosphate compounds to decrease the loss of the dielectric value, manipulate the relative permittivity with various temperatures, and improve the stability of the crystal structure in the high-temperature range.

The crystal structures of *M*_2_P_2_O_7_ compounds have been extensively investigated, and some metal pyrophosphates exhibit the allotropic property (a property of some compounds to exist in two or more crystal forms). For example, *β*-Ca_2_P_2_O_7_ is tetragonal, whereas *α*-Ca_2_P_2_O_7_ is monoclinic^7^. Ca_2_P_2_O_7_ is also an important material in the luminescence^8^ and biomaterial^9^ fields. The thortveitite form undergoes a reversible phase transformation below 600 °C from the *α*-form (occurring at low temperature) to the *β*-form (occurring at high temperature). However, the dichromate form undergoes irreversible transformation at temperatures above 700 °C. The thortveitite-form *M*_2_P_2_O_7_ (*M* = Mg^2+^, Mn^2+^, and Zn^2+^) compounds are difficult to sinter into dense ceramics^5^. SrZnP_2_O_7_, CaZnP_2_O_7_, *α*-Zn_2_P_2_O_7_, SrCuP_2_O_7_, Mn_2_P_2_O_7_, and CaCuP_2_O_7_ are effective glass-free low-temperature co-fired ceramic (LTCC) materials^2,10,11^. All these metal pyrophosphates react with silver (Ag), but CaZnP_2_O_7_ and SrZnP_2_O_7_ do not react with copper (Cu)^5^.

Unary metal pyrophosphate, such as Mg_2_P_2_O_7,_ was thermally synthesized by using minerals such as dittmarite (NH_4_MgPO_4_·H_2_O), struvite (NH_4_MgPO_4_·6H_2_O), and newberyite (MgHPO_4_·3H_2_O) as precursors^12^. Binary metal pyrophosphates, such as Mn_1.8_Co_0.2_P_2_O_7_, were synthesized from the thermal decomposition of manganese cobalt hydrogen phosphate trihydrate (Mn_0.9_Co_0.1_HPO_4_·3H_2_O)^13^. Another binary metal compound, CaCuP_2_O_7_, was synthesized by using a mixture of diammonium hydrogen phosphate ((NH_4_)_2_HPO_4_), calcium carbonate (CaCO_3_), and copper oxide (CuO) with the losses of carbon dioxide (CO_2_) and ammonia (NH_3_) gases based on the following equation (Eq. (1))^14^:

1 $${2}\left( {{\text{NH}}_{{4}} } \right)_{{2}} {\text{HPO}}_{{4}} \left( {\text{s}} \right) + {\text{CaCO}}_{{3}} \left( {\text{s}} \right) + {\text{CuO}}\left( {\text{s}} \right) \to {\text{CaCuP}}_{{2}} {\text{O}}_{{7}} \left( {\text{s}} \right) + {\text{3H}}_{{2}} {\text{O}}\left( {\text{g}} \right) + {\text{4NH}}_{{3}} \left( {\text{g}} \right) + {\text{CO}}_{{2}} \left( {\text{g}} \right)$$

To decompose the carbonate (CO_3_^2−^) and condense the phosphate (PO_4_^3−^), resulting in the formation of pyrophosphate (P_2_O_7_^4−^), the solid-state starting materials ((NH_4_)_2_HPO_4_ + CaCO_3_ + CuO) were homogeneously mixed first and kept at 700 °C. The obtained mixture was ground and then kept at 1060 °C for nine days. Using this thermal decomposition reaction, CaCuP_2_O_7_ was successfully synthesized. In addition, manganese cobalt magnesium hydrogen phosphate trihydrate (Mn_0.90_Co_0.05_Mg_0.05_HPO_4_·3H_2_O)^15^, manganese cobalt magnesium pyrophosphate dihydrate (Mn_1.8_Co_0.1_Mg_0.1_P_2_O_7_∙2H_2_O)^16^, and ammonium cobalt zinc manganese monohydrate (NH_4_Co_0.8_Zn_0.1_Mn_0.1_PO4·H2O)^17^ were employed as precursors to synthesize ternary metal pyrophosphates, namely, Mn_1.8_Co_0.1_Mg_0.1_P_2_O_7_, Mn_1.8_Co_0.1_Mg_0.1_P_2_O_7_, and Co_1.6_Zn_0.2_Mn_0.2_P_2_O_7_, respectively.

Most studies of different metal phosphate and metal pyrophosphate compounds have focused on both the syntheses and the characterizations of bulk^18,19^ and nano particles^20,21^, the kinetics and thermodynamics of the reaction^22,23^, and their properties^24,25^. For example, the photoluminescence of the LiMg_0.74_Mn_0.26_PO_4_ phosphor was investigated, and the results revealed that the luminescent property of this phosphor depended on its surface area^26^. Nevertheless, the relationship between crystal structures and dielectric properties is not widely understood. Therefore, the second aim of this work is to investigate the influence of the crystal structure on the dielectric phenomena of binary metal pyrophosphate compounds. Furthermore, substitutional solid solutions (binary metal compounds) based on the Hume-Rothery rules can be formed if the solute (Ca^2+^) and solvent (Cu^2+^ of Cu_2_P_2_O_7_) have similar valency (Cu = Ca = 2+) and the same crystal structure (*β*-Cu_2_P_2_O_7_ = *α*-Ca_2_P_2_O_7_ = monoclinic). This information shows a high possibility of substitutional metals between Cu and Ca ions forming a binary metal solid solution in pyrophosphate compounds, i.e., Ca_2−*x*_Cu_*x*_P_2_O_7_.

The dielectric properties of metal pyrophosphates occur due to two effects. They comprise the movement of *M*^2+^ ions in the *M*O_6_ octahedral and the shifting of O atoms in the collinear P−O−P bridge of the O_3_P−O−PO_3_ or P_2_O_7_^4−^ anion. If the collinear P−O−P bond of P_2_O_7_^4−^ is destroyed, some distortions will also occur in the MO6 octahedra. This phenomenon will improve the dielectric properties of materials by polarization production^27^. It is well known that the highly relative permittivity of BaTiO_3_ tetragonal perovskite occurs from the Ti^4+^ ion off-centered in the TiO_6_ octahedral.

The atomic radii of Cu^2+^ and Ca^2+^ are 0.73 and 1.00 Å, respectively, whereas their electronegativities are 1.90 and 1.00, respectively^28^. Doping the large cationic species, i.e., Ca^2+^, into the crystal structure of the Cu_2_P_2_O_7_ host resulted in the formation of Ca_2−*x*_Cu_*x*_P_2_O_7_ solid solutions. Both distortion of the *M*O_6_ octahedral and O shifting in the collinear P−O−P bond phenomena may occur. These phenomena may then improve the dielectric properties of Ca^2+^-doped Cu_2_P_2_O_7_ compounds at low sintering temperatures. Consequently, to investigate this doubt, this research synthesized Ca_2−*x*_Cu_*x*_P_2_O_7_ (*x* = 0.00−2.00) by using conventional and uncomplicated methods. Then, various scientific methods were used to characterize and confirm the synthesized Ca_2−*x*_Cu_*x*_P_2_O_7_ samples. Raman and Fourier transform infrared (FTIR) spectrophotometers were employed to characterize the vibrational spectra of the synthesized samples. X-ray diffraction (XRD) was used to investigate the crystal structure of the samples. The dielectric properties of the samples were also investigated by using an LCR meter, an effective technique for material measurement. The polarization phenomena in the crystal structure of the samples were studied to characterize the bond length and bond angle of Ca_2−*x*_Cu_*x*_P_2_O_7_. The chromaticity property was studied by applying the image sensor with a spatially multiplexed exposure-high dynamic range (SME-HDR) imaging function. The results were then compared to the CIE (International Commission on Illumination) chromaticity diagram (standard database). Consequently, these synthesized Ca_2−*x*_Cu_*x*_P_2_O_7_ compounds can be applied as effective optical materials. In addition, synchrotron light technology was also employed to analyze the Ca_2−*x*_Cu_*x*_P_2_O_7_ samples by using X-ray absorption spectroscopy (XAS) mode at the Cu and Ca *K* edges.

## Materials and methods

### Preparation

**Figure 1:**
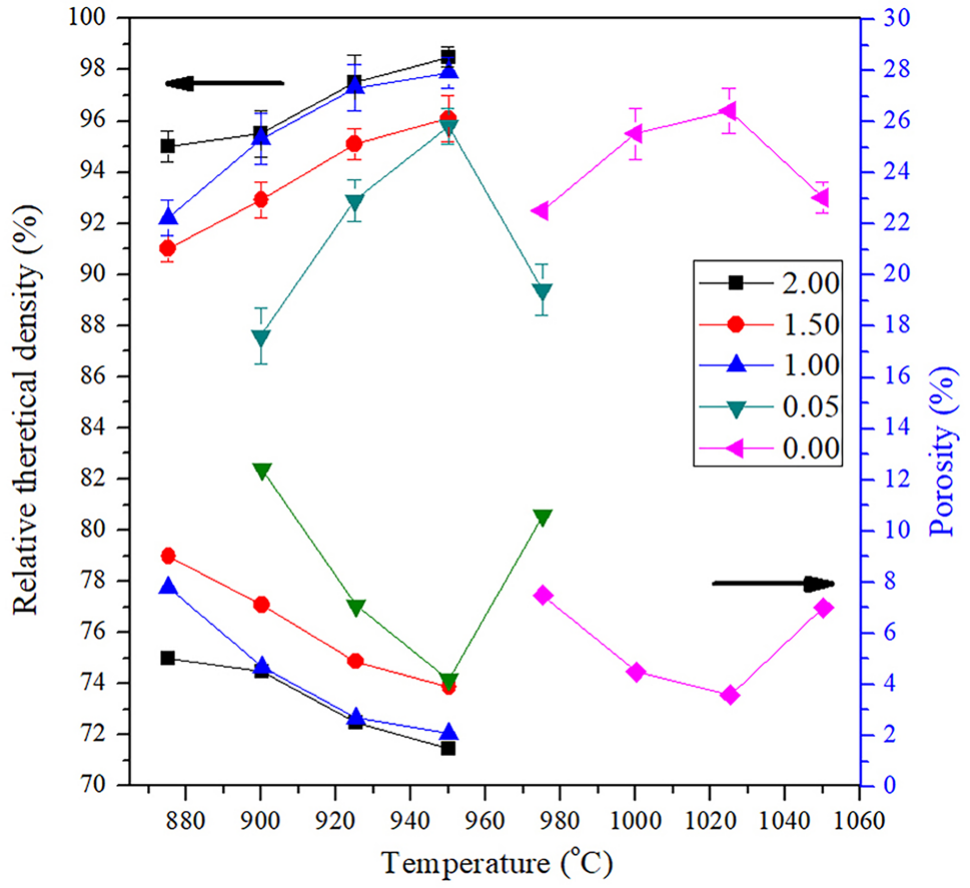
Relative theoretical densities (%) and porosities (%) of all prepared metal pyrophosphates (Ca_2−*x*_Cu_*x*_P_2_O_7_; *x* = 0.00−2.00).

Binary metal pyrophosphate samples with various Ca/Cu ratios (Ca_2−*x*_Cu_*x*_P_2_O_7_, *x* = 0.00, 0.50, 1.00, 1.50, and 2.00) were synthesized via the solid-state method. To avoid contamination, high-purity starting materials, namely, (NH_4_)_2_HPO_4_ (99%), CuO (99.9%), and calcium oxide (CaO, 99.9%), were selected in this preparation process. All starting materials were weighed according to the stoichiometric ingredients and then homogenized by vibratory milling with 10 mm spherical yttria (yttrium oxide, Y_2_O_3_)-stabilized zirconia (zirconium dioxide, ZrO_2_) (YSZ) grinding beads in ethanol media for 4 h. The dried powders were transferred to crucibles and directly heated at 1000 °C for 24 h for Ca_2−*x*_Cu_*x*_P_2_O_7_, when *x* = 0.00−1.50, and 800 °C for 24 h for Ca_2−*x*_Cu_*x*_P_2_O_7_, when *x* = 2.00. After that, the calcined powders were ball-milled anew, pressed uniaxially into small pellets at a pressure of 1000 kg·cm^−2^ and then sintered at 950 °C for 24 h for Ca_2−*x*_Cu_*x*_P_2_O_7_, when *x* = 0.00−1.50, and 1030 °C for 24 h for Ca_2−*x*_Cu_*x*_P_2_O_7_, when *x* = 2.00. The observed densities of all prepared metal pyrophosphates, in theory, were investigated by Archimedes’ principle and found to be in the range of 95−98% (Fig. 1). The preparation of the target powder samples (Ca_2−*x*_Cu_*x*_P_2_O_7_) was carried out according to the following reaction (Eq. (2)),

2 $$x{\text{CuO}}\left( {\text{s}} \right) + \left( {{2} - x} \right){\text{CaO}}\left( {\text{s}} \right) + {2}\left( {{\text{NH}}_{{4}} } \right)_{{2}} {\text{HPO}}_{{4}} \left( {\text{s}} \right) \to {\text{Ca}}_{{{2} - x}} {\text{Cu}}_{x} {\text{P}}_{{2}} {\text{O}}_{{7}} \left( {\text{s}} \right) + {\text{4NH}}_{{3}} \left( {\text{g}} \right) + {\text{3H}}_{{2}} {\text{O}}\left( {\text{g}} \right)$$

where *x* = 0.00−2.00.

### Characterization

The room temperature FTIR spectra of the samples were recorded by using a Perkin Elmer Spectrum GX FTIR spectrometer. The measured wavenumber range was 4000−400 cm^−1^, whereas the selected scan number and resolution were 8 scans and 4 cm^−1^, respectively. A Thermo Scientific DXR Raman microscope was used to record the Raman spectra in the Raman shift of 1300−100 cm^−1^ using a scan number of 8 scans. A Raman spectrum was observed by irradiating each synthesized sample with an intense beam of an argon ion (Ar^+^) laser with a wavenumber of 20,492 cm^−1^ (wavelength of 488 nm). The power of the incident beam was 12.5 mW. The XRD patterns of all prepared samples were recorded by using a D8 Advance X-ray diffractometer (XRD, Bruker AXS, Karlsruhe, Germany) with Cu K_α_ radiation (*λ* = 0.1546 nm) to analyze and confirm the crystal structures of the samples. The dielectric properties were analyzed as a function of the frequency (1−1000 kHz) and temperature (room temperature to 150 °C) using an Agilent/HP 4284A precision LCR meter (an effective technique for the material measurement with a wide frequency range (20 Hz−1 MHz) and superior signal performance to test materials to the most commonly used test standards). The Sony IMX214 CMOS image sensor (CIS, 13 MP “stacked” CIS with a spatially multiplexed exposure-high dynamic range (SME-HDR) imaging function) was applied to focus the colors of the samples. The results were then compared to the CIE (International Commission on Illumination) chromaticity diagram (standard database) to estimate the trend of the absorption wavelength. X-ray absorption spectroscopy (XAS) was performed at the Beamline 8 (BL8) Station of the National Synchrotron Research Center (NSRC, Nakhon Ratchasima, Thailand). BL8 of the NSRC is routinely operated for the XAS in an intermediate photon energy range from 1.25 to 10 keV^29^. The double crystal Ge(220) was used for the extended X-ray absorption fine structure (EXAFS) monochromator. The XAS spectra were detected in transmission mode at the copper (Cu) and calcium (Ca) *K*-edge.

### Ethics declarations

- The datasets generated and/or analyzed during the current study are not available in the other repository.
- The datasets used and/or analyzed during the current study available from the corresponding author on reasonable request.
- All data generated or analyzed during this study are included in this published article.
- The datasets generated and/or analyzed during the current study are not publicly available due [REASON WHY DATA ARE NOT PUBLIC] but are available from the corresponding author on reasonable request.
- The data that support the findings of this study are available from the corresponding author but restrictions apply to the availability of these data, which were used under license for the current study, and so are not publicly available. Data are however available from the authors upon reasonable request and with permission of the corresponding author.

## Results and discussion

### Structural, optical, and dielectric analyses

**Figure 2:**
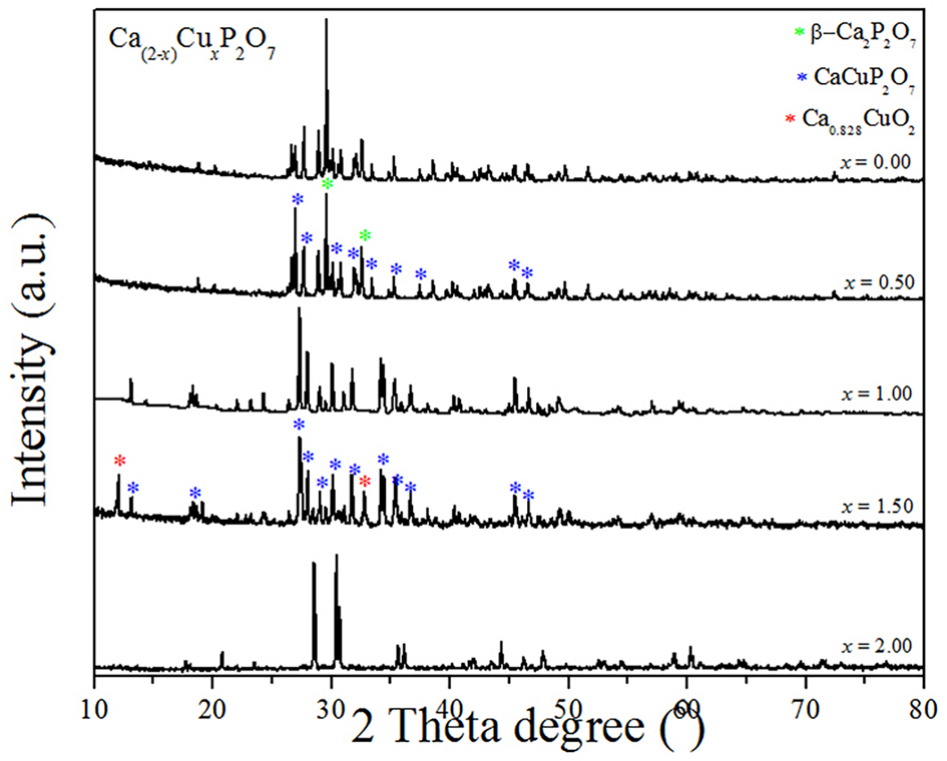
X-ray diffraction (XRD) patterns of the Ca_2−*x*_Cu_*x*_P_2_O_7_ powders (*x* = 0.00−2.00) synthesized from the solid-state method of homogenized (NH_4_)_2_HPO_4_, CuO, and CaO.

After applying the D8 Advance X-ray diffractometer, the resulting XRD patterns of the synthesized Ca_2−*x*_Cu_*x*_P_2_O_7_ powders (*x* = 0.00−2.00) are displayed in Fig. 2. The structures of Ca_2−*x*_Cu_*x*_P_2_O_7_ were analyzed through the Rietveld refinement analytic technique^30^ using the FullProf package^31^. A pseudo-Voigt function (a linear combination between the Lorentzian and Gaussian functions) was adequate at all times for obtaining good fits of the experimental data. The initial model for the refinement of the single phase structure (Ca_2_P_2_O_7_, CaCuP_2_O_7_ and Cu_2_P_2_O_7_) was taken from parameters described well in the Calvo research^32^.

**Figure 3:**
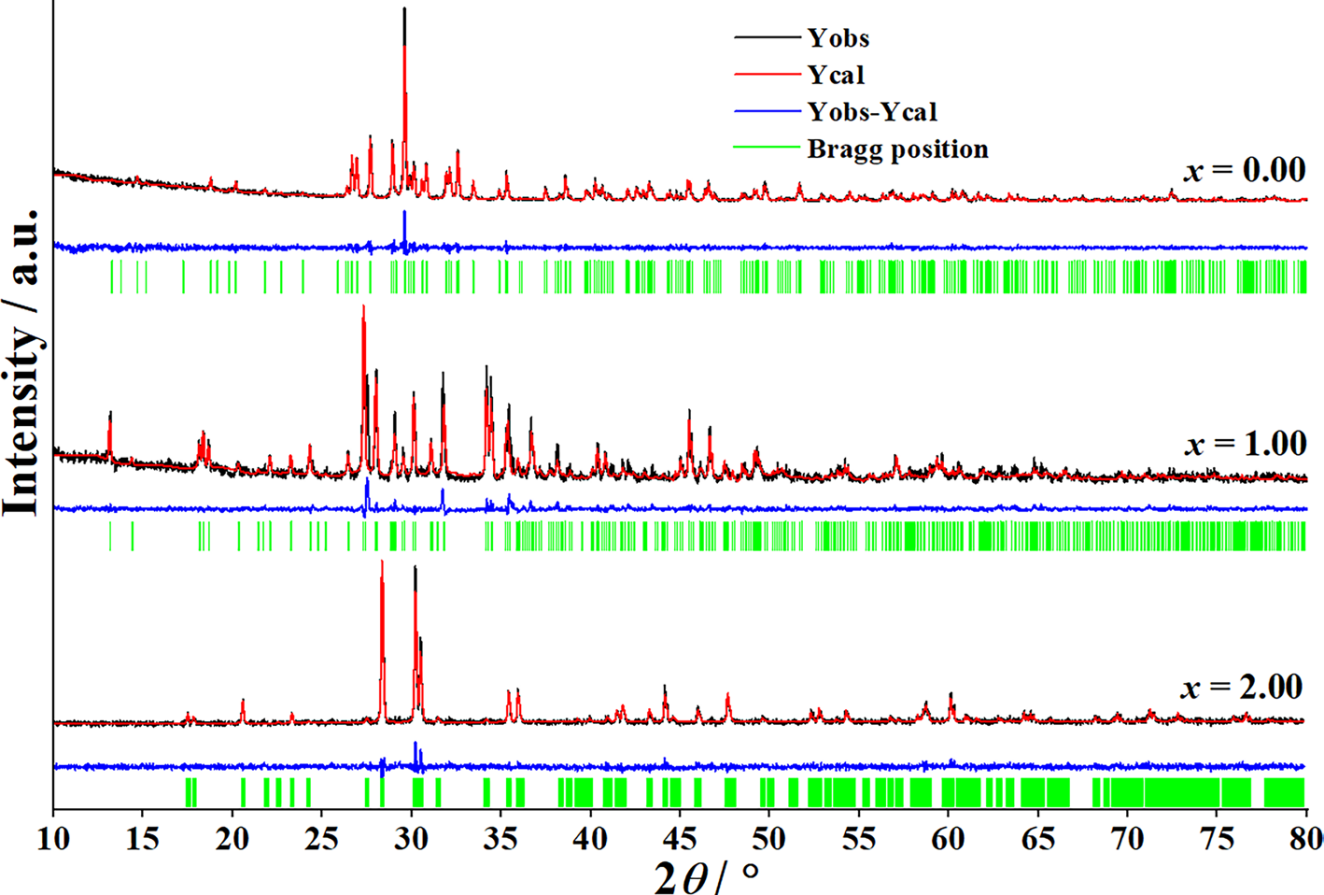
Rietveld refinement analytical results of the synthesized Ca_2−*x*_Cu_*x*_P_2_O_7_ samples when *x* = 0.00, 1.00, and 2.00.

In addition, Fig. 3 shows the corresponding Rietveld refinement results of Ca_2−*x*_Cu_*x*_P_2_O_7_ when *x* = 0.00, 1.00, and 2.00. Figure 3 shows the calculated (Y_cal_) and observed (Y_obs_) diffraction patterns as well as the different values between them (Y_obs_−Y_cal_) of the samples. The refinement plots gave the evolution of the XRD patterns in the various ratios between Ca and Cu (Ca_2−*x*_Cu_*x*_P_2_O_7_, *x* = 0.00, 1.00 and 2.00). The Rietveld refinement analysis and the XRD data of powders confirmed the formation of metal pyrophosphate compounds (Ca_2−*x*_Cu_*x*_P_2_O_7_).

The crystallographic information of the synthesized compounds is briefly described. When *x* = 0.00, the single metal pyrophosphate phase, *β*-Ca_2_P_2_O_7_, was obtained with the tetragonal crystal system, space group of *P4*_*1*_, space group number of 76, Schoenflies symbol of *C*_4_^2,33^, and number of formula units per unit cell or *Z* = 8. When *x* = 1.00, the binary metal pyrophosphate phase, CaCuP_2_O_7_, was obtained with the monoclinic crystal system, space group of *P2*_*1*_*/c*, space group number of 14, Schoenflies symbol of *C*_2h_^5^, and *Z* = 4. Finally, when *x* = 2.00, another single metal pyrophosphate phase, *α*-Cu_2_P_2_O_7_, was obtained with the monoclinic crystal system, space group of *C*2/*c*, space group number of 15, Schoenflies symbol of *C*_2h_^6^, and *Z* = 4. For other Ca_2−*x*_Cu_*x*_P_2_O_7_ samples, when *x* = 0.50, there were two phases between CuCaP_2_O_7_ and *α*-Cu_2_P_2_O_7_, whereas when *x* = 1.50, two phases between CuCaP_2_O_7_ and *β*-Ca_2_P_2_O_7_ were then observed. The P−O−P bond angles (of the O_3_P−O−PO_3_ bridge of P_2_O_7_^4−^) and *M*−O_6_ bond lengths (*M* = Ca or Cu) were determined by using refinement analysis, and the obtained values are summarized in Table 1.

**Table 1:** Bond angles and bond lengths from the Rietveld refinement analytic technique for the synthesized Ca_2−*x*_Cu_*x*_P_2_O_7_ samples; *x* = 0.00, 1.00, and 2.00.

Samples	POP Angle (°)	M-O_x_ bond length (Å)					SG	Z	R_p_	R_Wp_	R_exp_	χ^2^
		Bonding	No. 1	No. 2	No. 3	No. 4						
x = 2.00	154.60(10)	Cu-O_eq_	1.886(5)	–	–	–	*C*2/*c*	4	0.093	0.117	0.107	1.2
		Cu-O_eq_	1.942(6)	–	–	–						
		Cu-O_eq_	1.980(5)	–	–	–						
		Cu-O_eq_	1.995(5)	–	–	–						
		Cu-O_ax_	2.354(9)	–	–	–						
		Cu-O_ax_	2.920(9)	–	–	–						
x = 1.00	159.00(1)	Cu-O_eq_	1.853(3)	–	–	–	*P*2_1_/*n*	4	0.094	0.130	0.077	2.86
		Cu-O_eq_	2.004(2)	–	–	–						
		Cu-O_eq_	2.106(1)	–	–	–						
		Cu-O_eq_	2.129(5)	–	–	–						
		Cu-O_ax_	2.245(3)	–	–	–						
		Cu-O_ax_	2.811(1)	–	–	–						
x = 0.00	116.51(7) 140.96(1)	Ca-O1	2.53(5)	2.52(6)	2.55(4)	2.49(5)	*P*4_1_	8	0.107	0.144	0.126	1.31
		Ca-O2	2.33(4)	2.60(5)	2.65(6)	2.19(4)						
		Ca-O3	2.50(5)	2.89(4)	2.61(5)	2.36(5)						
		Ca-O4	2.80(7)	2.16(6)	2.26(10)	2.56(7)						
		Ca-O5	2.36(4)	2.27(4)	2.26(4)	2.28(4)						
		Ca-O6	2.18(6)	2.30(4)	2.34(5)	2.53(5)						
		Ca-O7	2.91(4)	2.83(5)	2.50(4)	2.81(5)						

where eq and ax subscripts are the equatorial and axial (or apical) positions, respectively, and ***χ***^**2**^ is the goodness of fit. *ND* is not detected.

X-ray absorption near-edge structure (XANES) is very sensitive to both the change in the local geometry (especially the ligand environment of the metal) and the oxidation state^34^. Therefore, the spectra were collected at both the Ca and Cu *K*-edges. They could help to understand the Fourier transform evolutions^34^. The X-ray absorption edge energies (*E*_0_) of the synthesized Ca_2−*x*_Cu_*x*_P_2_O_7_ compounds at the Ca and Cu *K*-edges are listed in Table 2.

**Table 2:** X-ray absorption edge energies (*E*_0_) of the synthesized Ca_2−*x*_Cu_*x*_P_2_O_7_ compounds when *x* = 0.00−2.00.

*x* values	Compounds	X-ray absorption edge energies (*E*_0_) / electron Volt, eV	
		Ca *K*-edge	Cu *K*-edge
Cu^0^	Cu^0^	ND	8978.45
Cu^1+^	Cu_2_O	ND	8979.52
Cu^2+^	CuO	ND	8987.72
Ca^2+^	CaO	4033.09	−
2.00	Cu_2_P_2_O_7_	ND	8987.88
1.50	Ca_0.5_Cu_1.5_P_2_O_7_	4033.14	8987.08
1.00	CaCuP_2_O_7_	4033.42	8987.09
0.50	Ca_1.5_Cu_0.5_P_2_O_7_	4033.42	8987.13
0.00	Ca_2_P_2_O_7_	ND	ND

*ND* is not detected.

**Figure 4:**
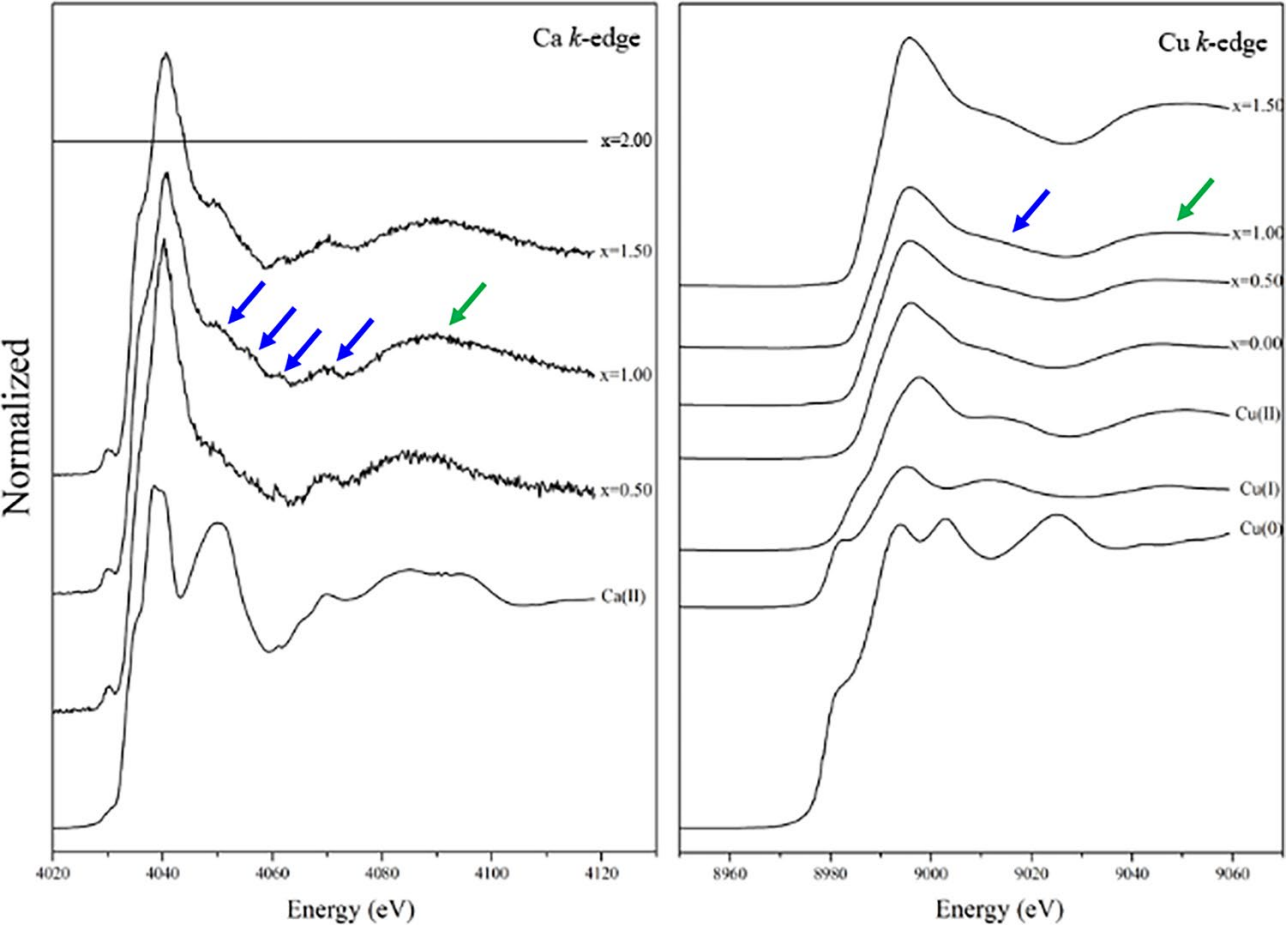
Local environment of Ca atoms when they entered the Cu_2_P_2_O_7_ structure, resulting in the formation of Ca_2−*x*_Cu_*x*_P_2_O_7_.

The *E*_0_ values of the various Cu valences (Cu^0^, Cu^1+^, and Cu^2+^) obtained in this work are in line with the information reported by Yano and Yachandra^34^. They reported that the *E*_0_ values increase with increasing oxidation state. They also described that an electron in an atom experiences the full charge of the positive nucleus. In contrast, in the case of many electrons, the electrons in an outer layer are simultaneously repelled by the negatively charged electrons and attracted to the positive nucleus. The lower the oxidation state of metals is, the less positive the overall charge of the atom. Consequently, to excite an electron from an orbital, more energy is required. In summary, when the metal has a more positive charge, the *E*_0_ values (XANES spectra) shift to a higher energy^34^. According to Table 2, in the Cu *K*-edge, the *E*_0_ values of the synthesized Ca_2−*x*_Cu_*x*_P_2_O_7_ samples (*x* = 0.50−2.00) were similar to the *E*_0_ values of Cu^2+^O, indicating that Cu^2+^ was monoclinic. In addition, the XANES spectra of samples in the Ca *K*-edge showed *E*_0_ values similar to Ca^2+^O, indicating that there was Ca^2+^ in the crystal structure of the Cu_2_P_2_O_7_ host, resulting in the formation of Ca_2−*x*_Cu_*x*_P_2_O_7_. Figure 4 presents the local environment of Ca atoms when they entered the Cu_2_P_2_O_7_ structure. The spectra of Ca and Cu in the CuCaP_2_O_7_ compound were different. These results demonstrated that the coordinated environments of the divalent Ca in CuCaP_2_O_7_ are significantly different^35^.

**Figure 5:**
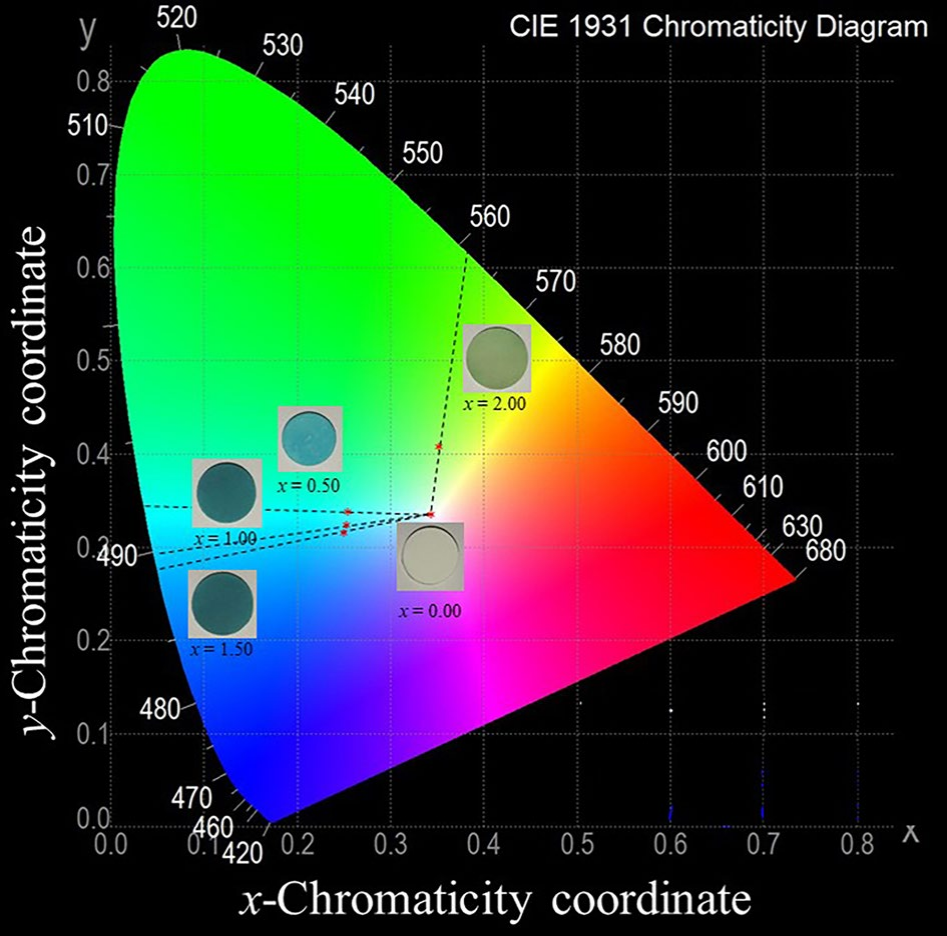
Optical properties and corresponding CIE (International Commission on Illumination) chromatic coordinates of Ca_2−*x*_Cu_*x*_P_2_O_7_; *x* = 0.00−2.00.

The coordinated complexes with different properties have different colors, such as blue for Cu(NH_3_)_4_H_2_O)_2_^2+^, red for Co(NH_3_)_5_H_2_O^3+^, and green for CoF_6_^3−^. This different color phenomenon was well explained by the crystal field theory (CFT) described by El Jazouli et al. and Chen et al.^36,37^ The optical properties and the corresponding CIE chromatic coordinates^36,38,39^ of Ca_2−*x*_Cu_*x*_P_2_O_7_ samples (*x* = 0.00−2.00) are shown in Fig. 5. All Ca/Cu ratio compounds, except the composition with *x* = 0.00 (Ca_2_P_2_O_7_), showed a greenish color, in which Ca_2_P_2_O_7_ exhibited a colorless powder. The colors of the samples were dictated by the elongation or compression of the *z* ligand bonds of the Cu^2+^ ion. The result of the composition with *x* = 2.00 (Cu_2_P_2_O_7_) illustrated a yellowish-green color, while the binary metal compounds (*x* = 0.50−1.50) presented color tones that changed from blue-green to bluish-green.

The mean static atomic dielectric constants (*ε*_r_) of the synthesized Ca_2−*x*_Cu_*x*_P_2_O_7_ compounds were estimated using the well-known Clausius-Mossotti relation^40^ as the following equation (Eq. (3)):

3 $$\varepsilon_{r} = \left( {\frac{{3V_{m} + 8\pi \alpha_{D} }}{{3V_{m} - 4\pi \alpha_{D} }}} \right)$$

where *α*_D_ is the sum of the dielectric polarizabilities of individual ions and *V*_m_ is the molar volume.

The effect of porosity on the permittivity was eliminated by applying Bosman and Havinga’s correction^41^ as shown in Eq. (4), which can be used for some materials, i.e., dense ceramics, having porosities lower than 5%:

4 $$\varepsilon_{{\text{r,corrected}}} = \varepsilon_{{\text{r,measured}}} \left( {{1} + {1}.{5}P} \right)$$

where *ε*_r,measured_ and *ε*_r,corrected_ are the measured and corrected relative permittivity, respectively, and *P* is the fractional porosity.

**Figure 6:**
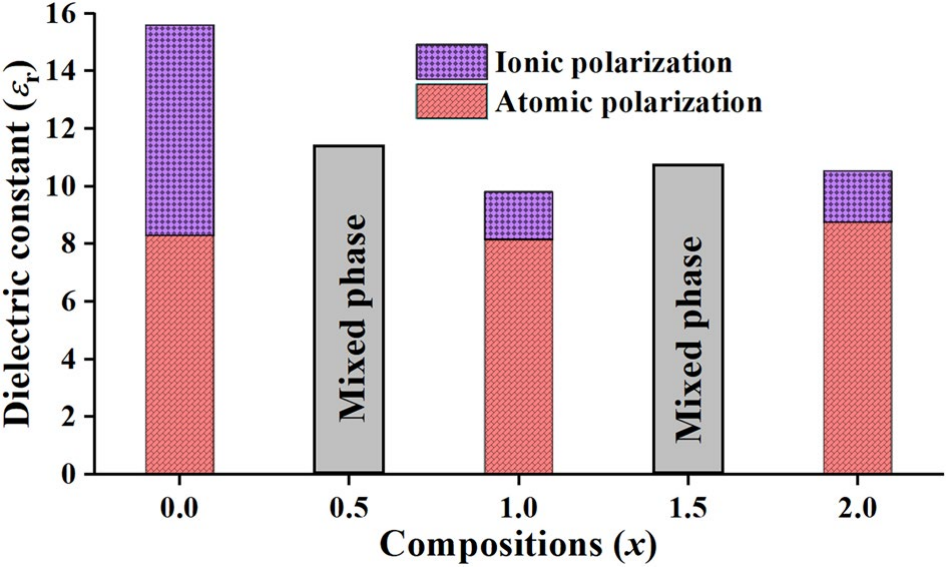
Dielectric constant as a function of the composition *x* of Ca_2−*x*_Cu_*x*_P_2_O_7_; *x* = 0.00−2.00.

After applying the Clausius-Mossotti relation (Eq. (3)), the dielectric constant (*ε*_r_) values as a function of the composition *x* of the synthesized Ca_2−*x*_Cu_*x*_P_2_O_7_ (*x* = 0.00−2.00) are presented in Fig. 6, which shows the combination values between the calculated data (atomic polarization part, red bars) and measured results (atomic polarization part + ionic polarization part, red and purple bars). The single metal pyrophosphates (Ca_2_P_2_O_7_ and Cu_2_P_2_O_7_) showed ε_r_ values of 15.6 and 10.5, respectively, which were higher than the *ε*_r_ value of binary metal pyrophosphates (i.e., CaCuP_2_O_7_, *ε*_r_ = 9.8). The ε_r_ values of the mixing phases of binary metal pyrophosphates (Ca_1.50_Cu_0.50_P_2_O_7_ and 1.50 (Ca_0.50_Cu_1.50_P_2_O_7_) have not been estimated because of the unknown amount of exact phase composition. The Clausius-Mossotti equation focused on only the dielectric constant from atomic polarization (electron cloud bias in electric fields). Indeed, the samples were measured at a frequency of 1 MHz for the decreasing extrinsic factor, and the polarization caused the movement of both cations (Cu^2+^, Ca^2+^, and P^5+^) and anions (O^2−^) in the crystal Ca_2−*x*_Cu_*x*_P_2_O_7_ structure. The movement of the ions in the electric field was caused by an increasing dielectric constant compared to the calculated data using the Clausius-Mossotti equation. The equation used in this study considered the dielectric constant, using the bond angle, bond length, and volume of the *M*O_6_ octahedra.

**Figure 7:**
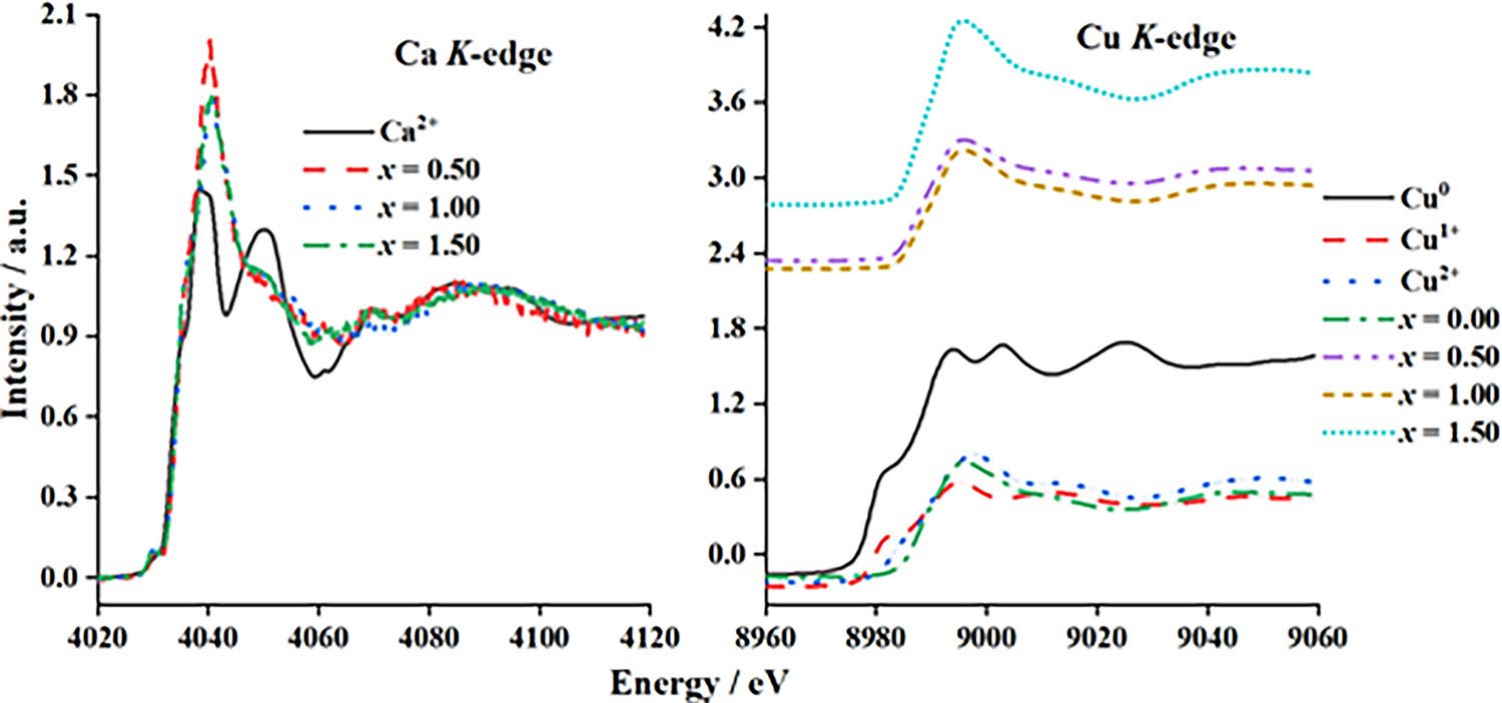
Extended X-ray absorption fine structure (EXAFS) spectra of Cu^0^, Cu^1+^, Cu^2+^, Ca^2+^, and Ca_2−*x*_Cu_*x*_P_2_O_7_; *x* = 0.00−1.50.

The extended X-ray absorption fine structure (EXAFS) spectra of the synthesized Ca_2−*x*_Cu_*x*_P_2_O_7_ samples are shown in Fig. 7. The environment around Cu atoms was investigated. The primitive EXAFS model was taken from parameters obtained from the Rietveld refinement of each sample.

Details of the EXAFS spectroscopic fitting of the Ca_2−*x*_Cu_*x*_P_2_O_7_ samples are summarized in Table 3, which shows the distortion of the CuO_6_ octahedra. The spectra of *x* = 0.00 were undetectable because of the limitation of the instrument in beamline 8 of the National Synchrotron Research Center (Thailand). As presented in Table 3, the samples, when *x* = 1.00 and 2.00, showed three main shells. The first shell of the spectrum from the model consisted of four equatorial oxygen atoms, Cu−O1_eq_, Cu−O2_eq_, Cu−O3_eq_, and Cu−O4_eq,_ of the CuO_6_ octahedral. Then, the second shell detected only one axial oxygen atom, Cu−O5_ax_. The last axial oxygen atom, Cu−O6_ax,_ was observed in the third shell. The Cu atoms of Cu−O6 were also combined with the phosphorus atom Cu−P. Different radial distances (R/Å) between the Rietveld refinement and EXAFS fitting may be the cause of the measurement type of each technique. X-ray diffraction (Rietveld refinement) was used to investigate the global structure, while X-ray absorption (EXAFS fitting) was used to probe the details of the Cu/Ca local structure^42,43^. The fitting statistic factor (*R*-factor) of *x* = 1.00 was worse than that of *x* = 2.00 because of two important factors. First, the crystal structure of *α*-CaCuP_2_O_7_ (*x* = 1.00) was less symmetric than that of another sample (Cu_2_P_2_O_7_ (*x* = 1.00)). Second, *α*-CaCuP_2_O_7_ (*x* = 1.00) exhibited four different types of atomic positions in the unit cell.

**Table 3:** Bond length from EXAFS fitting for Ca_2−*x*_Cu_*x*_P_2_O_7_ samples; *x* = 1.00 and 2.00.

x values	Compounds	Path	Shell	CN	*R* / Å	*σ*^2^ / Å^2^	*R*-factor
2.00	Cu_2_P_2_O_7_	Cu−O_eq_	1	2	1.90505	0.00508	0.00369
		Cu−O_eq_	1	2	1.96427	0.00499	
		Cu−O_ax_	1	1	2.30075	0.03223	
		Cu−O_ax_	2	1	2.91358	0.00796	
1.00	CaCuP_2_O_7_	Cu−O_eq_	1	1	1.88239	0.05892	0.01308
		Cu−O_eq_	1	2	1.94469	0.00529	
		Cu−O_eq_	1	1	1.99797	0.06324	
		Cu−O_ax_	1	1	2.15606	0.33359	
		Cu−O_ax_	2	1	2.88853	0.00839	

where eq and ax subscripts are equatorial and axial (or apical) positions, respectively. CN is the coordination number, *R* is the radial distance, *σ*^2^ is the mean squared displacement, and the *R*-factor is the fitting statistic factor.

### Vibrational spectroscopy

**Figure 8:**
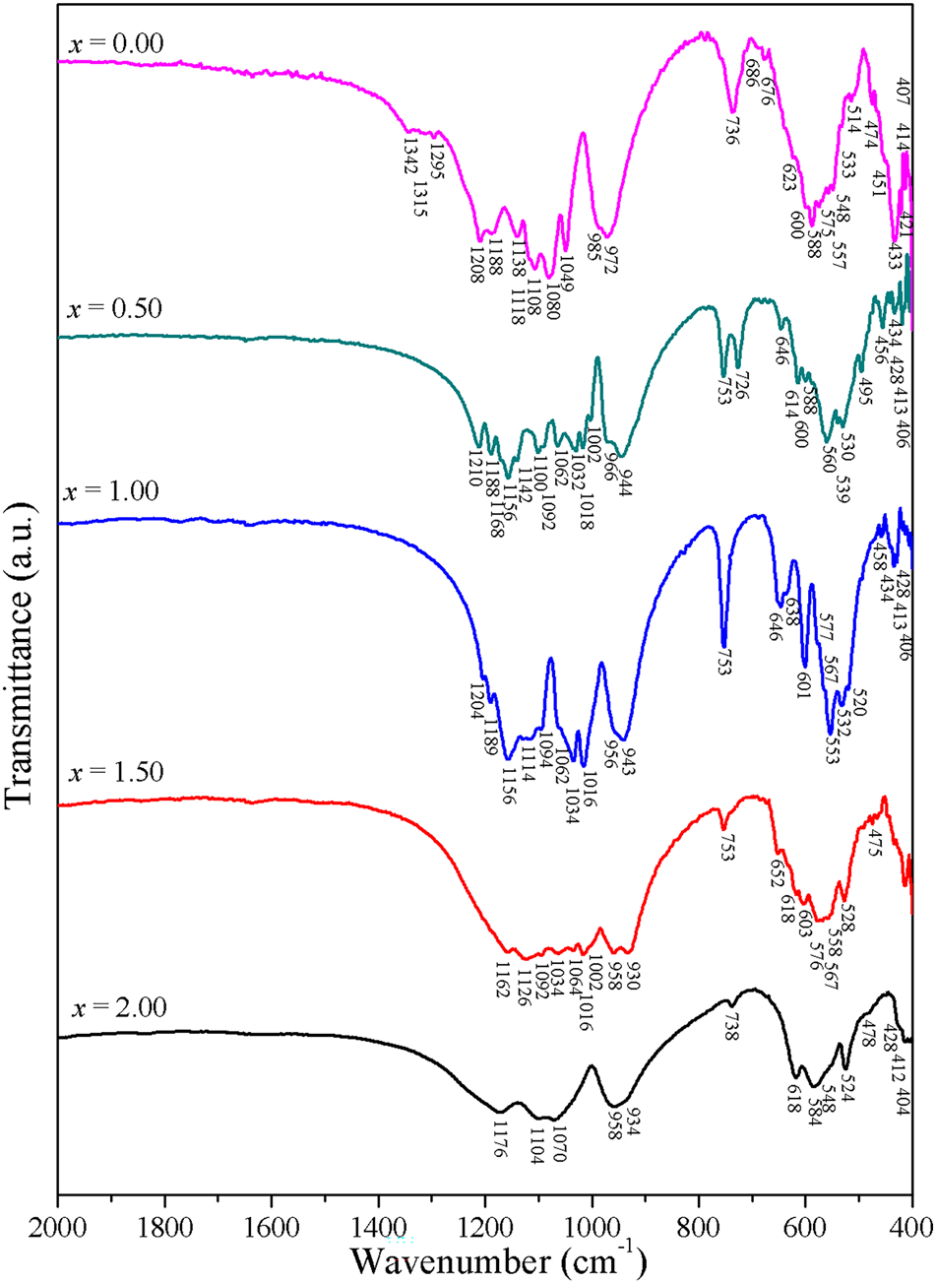
Fourier transform infrared (FTIR) spectra of the synthesized Ca_2−*x*_Cu_*x*_P_2_O_7_; *x* = 0.00−2.00.

FTIR and Raman spectroscopies are good methods for identifying the chemical bonding of rotational, vibration, and other low-frequency modes in the phosphate group^44^. After applying the Spectrum GX FTIR spectrometer, the FTIR spectra of the synthesized Ca_2−*x*_Cu_*x*_P_2_O_7_ samples are presented in Fig. 8, whereas the corresponding assignments are tabulated in Table 4. The FTIR spectra observed in this research are similar to the spectral results reported in the literature^12,13,15–17,45^. They successfully synthesized and investigated the vibrational spectroscopy of various single, double, and triple metal pyrophosphates, i.e., Mg_2_P_2_O_7_, Mn_1.8_Co_0.2_P_2_O_7_, Mn_1.8_Co_0.1_Mg_0.1_P_2_O_7_, Co_1.6_Zn_0.2_Mn_0.2_P_2_O_7_, and CoFeP_2_O_7_.

**Table 4:** Vibrational positions (wavenumber / cm^−1^) and vibrational assignments (modes) of the synthesized Ca_2−*x*_Cu_*x*_P_2_O_7_ samples obtained from the FTIR and Raman techniques.

Compounds	Wavenumber/cm^−1^	FTIR	Raman	Assignments
Ca_2−*x*_Cu_*x*_P_2_O_7_; *x* = 0.00−2.00	1250−1200	NO	Weak	*α*-phase characteristics
	1200−1100	Very strong	Very strong	*ν*_as_ PO_3_
	1100−1050	Very strong	Very strong	*ν*_s_ PO_3_
	1050−1000	Very strong	Very strong	*ν*_as_ PO_3_
	980−950	Strong	Very weak	*ν*_as_ P−O−P
	760−730	Medium	Weak	*ν*_s_ P−O−P
	650−280	Strong	Strong	*δ* OPO + *ν*_s_ *M*−O
	260−160	NO	Weak	*ν*_s_ *M*−O
	200−100	NO	Medium	Lattice vibration

*NO* is not observed.

The vibrational characteristics of the synthesized Ca_2−*x*_Cu_*x*_P_2_O_7_ are described in detail. The strong vibrational bands at approximately 1190 and 1060 cm^−1^ were attributed to an asymmetric (*ν*_*a*s_ PO_3_) vibrational mode of the PO_3_ unit of the pyrophosphate (O_3_P−O−PO_3_^4−^ or P_2_O_7_^4−^) ions, whereas a vibrational band at approximately 1100 cm^−1^ was attributed to the symmetric stretching (*ν*_s_ PO_3_) of the PO_3_ unit. The asymmetric (*δ*_as_ PO_3_) and symmetric (*δ*_s_ PO_3_) bending modes are observed at the vibrational positions at approximately 580 and 540 cm^−1^, respectively. The asymmetric (*ν*_as_ P−O−P) and symmetric stretching (*ν*_s_ P−O−P) modes of the P−O−P bridge of the O_3_P−O−PO_3_^4−^ group were observed at vibrational positions of approximately 960 and 740 cm^−1^, respectively. However, in the case of the Ca_2−*x*_Cu_*x*_P_2_O_7_ samples with *x* = 0.50 (Ca_1.5_Cu_0.5_P_2_O_7_) and *x* = 1.5 (Ca_0.5_Cu_1.5_P_2_O_7_), the P−O−P symmetric stretching mode appeared as two peaks in the range of 776−693 cm^−1^, which corresponded to the vibrational characteristics (symmetric stretching) of the P−O−P bridge. These detected peaks may be due to the mixing phases of the metal pyrophosphate compounds, i.e., Ca_2_P_2_O_7_ and CuCaP_2_O_7_. In addition, the rocking mode of the P−O−P deformations and the torsional and external modes were found in the 450−410 cm^−1^ regions.

**Figure 9:**
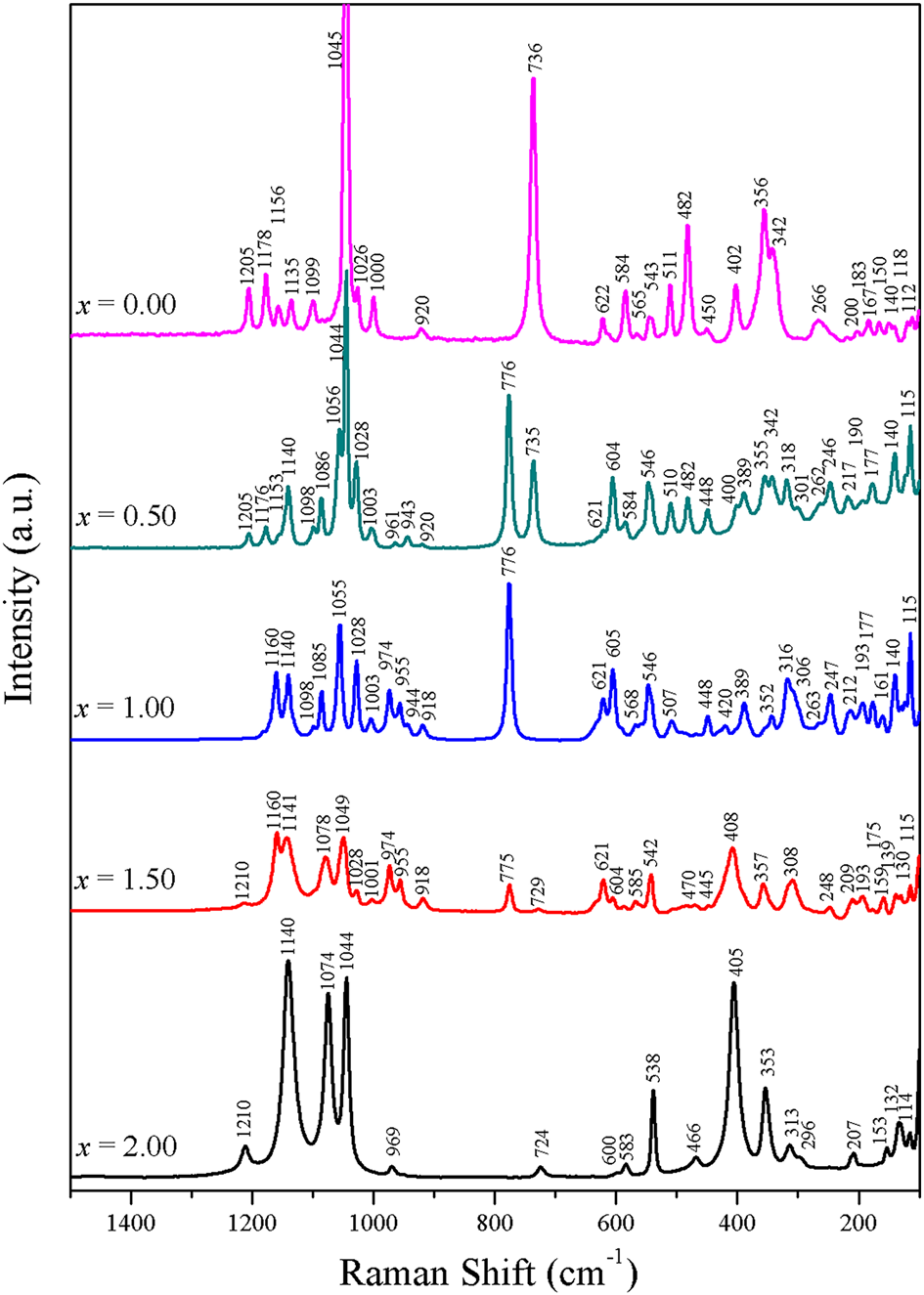
Raman spectra of the synthesized Ca_2−*x*_Cu_*x*_P_2_O_7_ compounds when *x* = 0.00−2.00.

The Raman spectroscopic technique was additionally applied to investigate and support the FTIR results, especially the vibrational spectroscopy of the metal oxide (*M*−O) bond as well as the lattice vibration by observation in the low frequency range (650−100 cm^−1^). Furthermore, the phase characteristics (*α*-, *β*-phases) of the metal pyrophosphate compounds can be observed from this spectroscopic technique. After applying the DXR Raman microscope, the Raman spectra of the samples are shown in Fig. 9, and the corresponding vibrational assignments are listed in Table 4. It was observed that the result corresponded well to the FTIR result. The Raman results showed the specific phase, which formed at high temperature in pyrophosphate with *x* = 1.00 (CaCuP_2_O_7_), as described in the literature^46^ through an undetectably weak peak at approximately 1210 cm^−1^. The three distinct peaks of Ca_2−*x*_Cu_*x*_P_2_O_7_, where *x* = 0.00, 0.50, 1.50 and 2.00, which originated from the *ν*_as_ PO_3_ vibrational characteristics, were observed and found to be at approximately 1210, 1140 and 1080 cm^−1^. The Raman spectra clearly showed that the studied metal pyrophosphates displayed sharpness and splitting, especially in the investigated frequency region (1300−100 cm^−1^). The vibrational analysis of the P_2_O_7_^4−^ ion, which contained the O−P−O radical (PO_2_^−^ of O_2_O−P−OPO_3_^4−^) and the P−O−P bride (of O_3_P−O−PO_3_^4−^), was exhibited in the Raman spectra. Moreover, *M*−O stretching and phase characteristics were also observed. The Raman spectra observed in this research were similar to the spectra reported by Sronsri et al.^12,13,15–17^ and Boonchom et al.^45^ The strong vibrational band at approximately 1100 cm^−1^ was attributed to the stretching of the PO_3_ unit of O_3_P−O−PO_3_^4−^. The asymmetric (*ν*_asym_ POP) and symmetric (*ν*_sym_ POP) stretching types of the P−O−P bridge of O_3_P−O−PO_3_^4−^ were detected at approximately 960 and 730 cm^−1^, respectively. The asymmetric (*δ*_asym_ PO_3_) and symmetric (*δ*_sym_ PO_3_) vibrational bending modes of O_3_P−O−PO_3_^4−^ were observed at approximately 600 and 520 cm^−1^, respectively.

### Dielectric and optical properties

#### Structural-dielectric relation

The bond angle and bond length were successfully investigated by using the Rietveld refinement technique, as shown in Table 1. The obtained refinement results were then used to describe the phenomena of the dielectric properties of the samples. In general, the dielectric properties of the metal pyrophosphate (*M*_2_P_2_O_7_) group occurred from two important effects, which consisted of O-atom shifting in the collinear P−O−P bridge and *M*^2+^-ion movement in the *M*O_6_ octahedral. According to previous works, due to the shifting of the O atom in the collinear P−O−P bridge, the P−O−P bond angles of Ca_2_P_2_O_7_ and Cu_2_P_2_O_7_ of 130°^47^ and 157°^48^ were reported, respectively. In this section, only three synthesized Ca_2−*x*_Cu_*x*_P_2_O_7_ samples, when *x* = 0.00, 1.00, and 2.00, were considered. The sample, when *x* = 0.00 (Ca_2_P_2_O_7_), showed two different P−O−P bond angles. First, a bond angle of 116.52° appeared for 4 clusters per unit cell with asymmetric P−O bond lengths of 1.765 Å and 1.887 Å. Second, a P−O−P bond angle of 140.96° appeared for 4 clusters per unit cell with symmetric P−O bond lengths of 1.536 Å and 1.827 Å. The sample, when *x* = 2.00 (Cu_2_P_2_O_7_), had a P−O−P bond angle of 154.6° and 4 clusters per unit cell with a symmetric P−O bond length of 1.574 Å. Pogorzelec-Glaser et al.^46^ reported that at high temperature, the binary metal pyrophosphate (Cu*M*^2+^P_2_O_7_) compounds crystallized in a monoclinic crystal system with the space group of *C*2/*m*, and the P−O−P angle was linear (180°). The sample, when *x* = 1.00 (CaCuP_2_O_7_), exhibited the space group of *P*2_1_/*n*. The refinement result showed a P−O−P bond angle of 159.00° and 4 clusters per unit cell with asymmetric P−O bond lengths of 1.592 Å and 1.521 Å. However, the number of P−O−P clusters in each Ca_2−*x*_Cu_*x*_P_2_O_7_ sample (*x* = 0.00, 1.00, and 2.00) was equal (4). Based on these obtained results, the P−O−P cluster number did not affect the polarization of the samples.

The single metal pyrophosphate, when *x* = 0.00 (Ca_2_P_2_O_7_), showed an outstanding dielectric constant (15.6, as shown in Fig. 6). This was a very high polarization; it therefore caused and made the narrow P−O−P bond angle. In addition, the long P−O bond length of the sample of *x* = 0.00 (Ca_2_P_2_O_7_), resulting in weak bonding, was better than the samples of *x* = 1.00 (CaCuP_2_O_7_) and *x* = 2.00 (Cu_2_P_2_O_7_). Additionally, the volume of the octahedral coordination was calculated using the method reported by Swanson et al.^49^ to present the relationship between the polarization and metal oxide bonding. In addition, the distortion index (*D*) was used to describe the distortion of the sample crystal structure. Baur^50^ described the calculation of the *D* value based on the bond lengths, as shown in Eq. (5).

5 $$D = \frac{1}{n}\sum\limits_{i = 1}^{n} {\frac{{\left| {l_{i} - l_{av} } \right|}}{{l_{av} }}}$$

where *l*_av_ is the average bond length and *l*_*i*_ is the atomic distance from the central atom to the *i*th coordinating atom.

The refinement analysis results also showed a change in the average *M*−O bond lengths in the *M*O_6_ octahedral site, which caused molecular polarization. As demonstrated in Table 5, both the average bond lengths and octahedral volumes decreased with increasing *x* values. However, a different result was observed for the distortion index. The distortion index values increase with increasing *x* values, which then decreases the molecular polarization, resulting in a decrease in the dielectric constant (*ε*_r_). These analyses showed that the polarization of Ca_2−*x*_Cu_*x*_P_2_O_7_ occurred due to O shifting in the collinear P−O−P bridge, which is the main factor in the generation of a narrow bond angle that causes high polarization and a high dielectric constant. Moreover, the movement of *M*^2+^ ions in the *M*O_6_ octahedral was a supplementary factor, in which the longer average *M*−O bond length and larger octahedral volume led to the high polarization and high dielectric constant of the materials.

**Table 5:** Average bond length, octahedral volume, and distortion index of Ca_2−*x*_Cu_*x*_P_2_O_7_ samples (*x* = 0.00, 1.00, and 2.00).

*x* values	Compounds	Average bond lengths/Å	Octahedral volumes/Å^3^	Distortion index
2.00	Cu_2_P_2_O_7_	2.1794	12.6198	0.1400
1.00	CaCuP_2_O_7_	2.4032	15.9050	0.0886
0.00	Ca_2_P_2_O_7_	2.4479	18.6696	0.0655

#### Structural-optical relation

**Figure 10:**
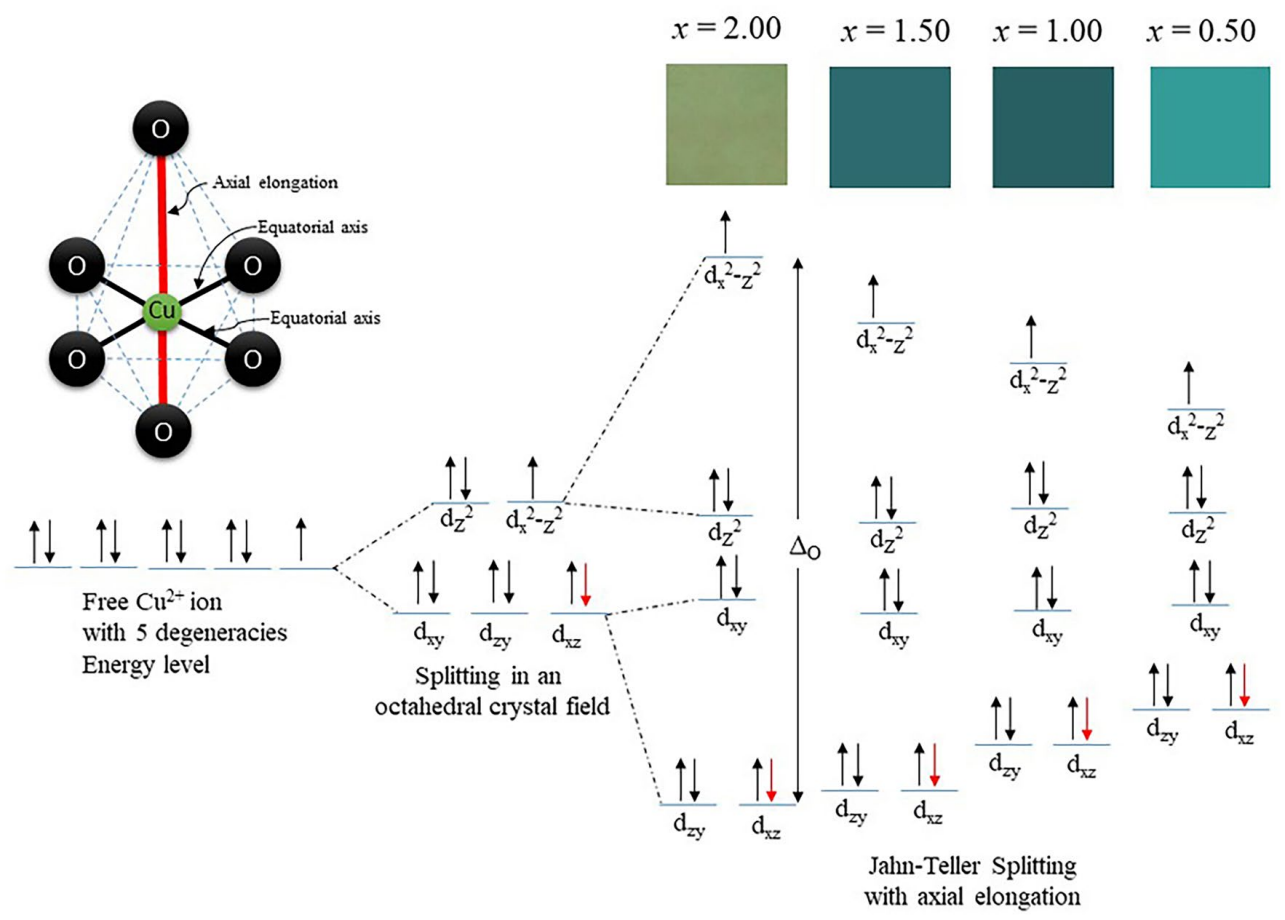
Octahedral splitting diagram of the synthesized Ca_2−*x*_Cu_*x*_P_2_O_7_ samples; *x* = 0.50−2.00.

The distortion of the *M*O_6_ octahedral can increase the Cu−O_6_ bond lengths of Ca_2−*x*_Cu_*x*_P_2_O_7_, resulting in an increase in the octahedral crystal field splitting energy (Δ_0_, please see Fig. 10). The Δ_0_ values of the synthesized Ca_2−*x*_Cu_*x*_P_2_O_7_ samples (*x* = 0.50−2.00) are listed in Table 6.

**Table 6:** Approximate wavelength of the energy absorption.

*x* values	Compounds	Wavelengths/nm		Δ_0_/kJ·mol^−1^	Cu−O_ax_ bond lengths/Å	
		Observed	Absorbed		XRD	EXAFS
2.00	Cu_2_P_2_O_7_	561	403	297	2.92	2.91
1.50	Ca_0.5_Cu_1.5_P_2_O_7_	488	642	186	ND	ND
1.00	CaCuP_2_O_7_	489	644	185	2.81	2.89
0.50	Ca_1.5_Cu_0.5_P_2_O_7_	492	648	184	ND	ND
0.00	Ca_2_P_2_O_7_	ND	ND	ND	ND	ND

*ND* is not detected.

As presented in Table 6, Δ_0_ increased with increasing Cu^2+^ fraction in the Ca_2−*x*_Cu_*x*_P_2_O_7_ compound, and when *x* = 2 (Cu_2_P_2_O_7_), the highest Δ_0_ value was obtained. The compounds illustrated the change in color from blue-green to bluish-green. The colorless compound, when *x* = 0.00 (Ca_2_P_2_O_7_), was due to the fulfillment state in the octet rule of Ca^2+^ ions in the structure, despite the distortion appearing in the CaO_6_ octahedral site. The octahedral splitting diagram of Ca_2−*x*_Cu_*x*_P_2_O_7_; *x* = 0.50−2.00 is summarized and presented in Fig. 10. Total interpretations showed that the *M*O_6_ octahedral distortion affected both the color of the sample and the polarization of the octahedral unit, as reflected in the dielectric constant of the compounds.

## Conclusions

Binary metal pyrophosphates (Ca_2−*x*_Cu_*x*_P_2_O_7_) were successfully synthesized via a solid-state reaction process. The synthesized Ca_2−*x*_Cu_*x*_P_2_O_7_ samples were systematically characterized by various scientific instruments. The structural analysis exhibits the single solid phase for the obtained Ca_2_P_2_O_7_, CaCuP_2_O_7_, and Cu_2_P_2_O_7_ samples and the mixing solid phases for the obtained Ca_1.5_Cu_0.5_P_2_O_7_ and Ca_0.5_Cu_1.5_P_2_O_7_ samples. The tetragonal crystal system with the *P4*_*1*_ space group is a crystal for *β*-Ca_2_P_2_O_7_, while the monoclinic crystal systems with the *P2*_*1*_*/c* and *C*2/*c* space groups are crystals for CaCuP_2_O_7_ and *α*-Cu_2_P_2_O_7_, respectively. The color of the samples changed from yellowish-green to bluish-green when the Cu content increased because the absorption wavelength increased and corresponded to a decrease in the z-axis expansion. Using the Rietveld refinement method, the P–O–P bond angle and P–O bond length and details of the octahedral MO_6_ (the average bond length, octahedral volume, and distortion index) were calculated. The addition of Cu^2+^ ions in the Ca_2_P_2_O_7_ structure resulting in distortion of the crystal structure affected the changes in the bond length and bond angle of the P–O–P groups in the P_2_O_7_^4−^ ions and the octahedral volume and average bond lengths in the octahedral MO_6_ site. Shifting O atoms in the collinear P–O–P bridge (a narrow bond angle) and the movement of M^2+^ ions in octahedral MO_6_ (the longer average M–O bond length and larger octahedral volume) are probably the main factors leading to the high values of polarization and dielectric constant of metal pyrophosphates. Finally, these results illustrated that the distortion of the octahedral MO_6_ resulted in a straightway effect on the color of the metal pyrophosphate compounds, while the change in the P–O–P bridge influenced the dielectric properties.
